# Three-dimensional free-standing gold nanowire networks as a platform for catalytic applications†

**DOI:** 10.1039/d2ra08035d

**Published:** 2023-02-03

**Authors:** Mohan Li, Nils Ulrich, Ina Schubert, Wilfried Sigle, Michael Florian Peter Wagner, Christina Trautmann, Maria Eugenia Toimil-Molares

**Affiliations:** a Materials Research, GSI Helmholtzzentrum für Schwerionenforschung 64291 Darmstadt Germany M.E.ToimilMolares@gsi.de; b Materials and Earth Sciences, Technische Universität Darmstadt 64287 Darmstadt Germany; c Max Planck Institute for Solid State Research Heisenbergstr. 1 70569 Stuttgart Germany

## Abstract

We report the catalytic performance of networks of highly interconnected Au nanowires with diameters tailored between 80 and 170 nm. The networks were synthesized by electrodeposition in etched ion-track polymer templates, and the synthesis conditions were developed for optimal wire crystallinity and network homogeneity. The nanowire networks were self-supporting and could be easily handled as electrodes in electrochemical cells or other devices. The electrochemically active surface area of the networks increased systematically with increasing the wire diameter. They showed a very stable performance during 200 CV cycles of methanol oxidation reactions, with the peak current density reaching up to 200 times higher than that of a flat reference electrode, with only a 5% drop in the peak current density. The Au nanowire networks proved to be excellent model systems for investigation of the performance of porous catalysts and very promising nanosystems for application in direct alcohol fuel cell catalysts.

## Abstract 2

Highly interconnected Au nanowire networks synthesized by electrodeposition in etched ion-track polymer templates show promising catalytic performance for methanol oxidation reaction.

## Graphical abstract

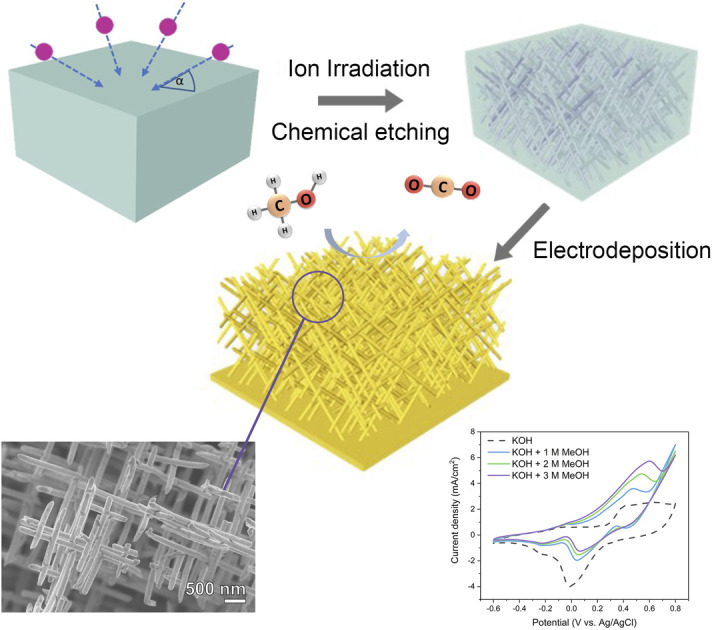

## Introduction

Nanostructured porous materials with enhanced surface areas are of increasing interest in various research fields, such as chemical and biological sensing, drug delivery, electrocatalysis, and gas storage.^1–5^ For many applications, nanostructured porous gold (Au) has become of special interest because of its various advantages, such as its biocompatible nature, electrical conductivity, optical properties,^6–8^ and its ability to act as a support for functionalization with carbohydrates, enzymes, DNA molecules, or thiols.^9,10^ While bulk gold is in general considered an inert metal, nanostructured gold has shown remarkable catalytic activity ascribed to its largely increased specific surface area for nanoscale structures. Examples uses include the oxidation of carbon monoxide (CO), alcohols, aldehydes, amines, and hydrocarbons.^11–17^

Heterogeneous catalysis and electrosynthesis are vital processes in the search for alternative and environmentally friendly sustainable energy.^18,19^ Nanostructured Au is expected to enable a more environmentally friendly electrochemical reaction process by acting as a catalyst during the selective oxidation of methanol, without the use of CO gas.^20–23^ Wittstock *et al.* demonstrated the formation of methyl-formate using dealloyed nanoporous Au as a catalyst during methanol oxidation with >97% selectivity.^24^

The selective oxidation of methanol can play a central role in future fuel cells, which are attracting increasing attention as power sources for portable devices since methanol possesses the advantages of easier transportation, more stable storage, and a higher energy density than hydrogen.^25–27^ Much research has been devoted to developing efficient catalysts for the methanol electro-oxidation process.^28–30^ For example, in direct methanol fuel cells (DMFs), nanoparticles such as carbon-supported platinum (Pt) and Pt–Ru nanoparticles^31^ are used as catalysts. Traditionally, Pt and Pt-based alloys are the preferred candidates as a catalyst for various electrochemical reactions, but for direct alcohol fuel cells, their low reaction rate and stability are raising some concerns regarding their long-term development.^32,33^ Pt-based catalysts also suffer from a severe CO poisoning effect, which can block the active reaction sites, due to the intermediates generated during the methanol electro-oxidation reactions.^34,35^ In contrast, Au exhibits no poisoning effect during the catalytic reaction process.^36,37^

In addition, the durability of nanoparticle-based catalysts is limited due to coalescence effects,^30^ which can result in a decrease in surface area and degradation of their catalytic performance. The development and investigation of nanostructured electrodes, which can avoid coalescence and maintain their morphology during the reaction, are of the utmost scientific and technological relevance. In this context, nanoporous Au (NPG) obtained by dealloying is considered a Pt-free and support-free alternative catalyst material. However, morphological changes and a decrease in the electrochemically active surface area (ECSA) were reported during the production of syngas with NPG acting as an electrode.^7,38^

Free-standing three-dimensional (3D) nanowire networks with high interconnectivity are a promising alternative for high activity catalysis, providing excellent mechanical stability and a large surface area in comparison to bulk materials and thin films. In this study, we produced nanowire networks by the electrodeposition of Au in ion-track etched polymer templates. The technique enables a precise control of the nanowire size, interconnectivity, and composition.^39,40^ By adjusting the wire parameters, the surface area can be tailored to provide an optimal reactive area for the catalysis of chemical reactions with highly improved reaction rates.^41–43^ Due to the enlarged surface area and often complex geometries of porous systems, the ECSA differs from the geometrical area. To characterize their catalytic ability, it is thus mandatory to accurately determine their ECSA.^44,45^ Previous studies reported the determination of the ECSA of porous Au electrodes by various methods, including impedance spectroscopy, double-layer capacitance measurements, and cyclic voltammetry studies.^46,47^

The diameter of the nanowires in the network was adjusted between 80 and 170 nm. The nanowires were grown in four different directions, resulting in highly interconnected nanowire networks with geometrical surface areas up to 50 times higher than the corresponding flat electrode. The ECSAs were determined by integrating the faradaic charge of monolayer Au oxide reduction, and were in good agreement with the estimated geometrical surface areas, demonstrating that the entire network took part in the electrochemical reaction. In addition, compared to nanoparticle-based electrodes, the mechanical stability of these 3D nanowire networks and the adjustable interspace between the wires guarantee sufficient access for reactants and products to the electrochemically active interface.

## Experimental

**Fig. 1:**
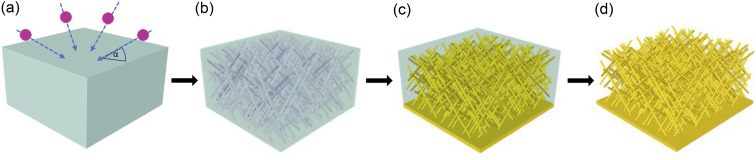
Schematic of nanowire network fabrication: (a) a PC polymer foil is irradiated with swift heavy ions (indicated by arrows) from four different directions, in each case at an angle *α* = 45° to the foil surface, (b) chemical etching of the generated ion tracks leads to the formation of a 3D nanochannel network, (c) fabrication of Au back electrode and electrodeposition of Au inside the nanochannels, and (d) removal of the polymer matrix to obtain a freestanding 3D nanowire network.

The synthesis of the nanowire networks required several process steps, including (i) the fabrication of porous polymer membranes by ion irradiation and track etching, (ii) the electrodeposition of gold into the nanochannels of the membrane, and (iii) release of the wire network by template dissolution (Fig. 1).

(i) The etched ion-track membranes with interconnected cylindrical nanochannels were fabricated by irradiating 30 μm thick polycarbonate (PC) foils (Makrofol N, Bayer AG) with Au ions with ∼2 GeV kinetic energy. The irradiations were conducted at the UNILAC linear accelerator of the GSI Helmholtz Centre for Heavy Ion Research in Darmstadt (Germany). Each ion creates a highly localized damaged region called an ion track that can be converted into an open channel by selectively removing the damage in an etching process. This leads to parallel aligned channels with randomly distributed distances. To achieve a network of interconnected channels, the films were irradiated from four directions, each under an angle of 45° to the film surface. After each irradiation step applying an ion fluence of 4 × 10^8^ ions per cm^2^, the film was rotated by 90° (see Fig. 1(a)). Before etching, the irradiated polymer foils were exposed to ultraviolet (UV) light (30 W, T-30M, Vilber Lourmat) from both sides, each for 1 h. This treatment is known to sensitize the ion tracks, resulting in a very narrow nanochannel diameter distribution.^48,49^ For track etching, we used an aqueous alkaline solution of 6 M NaOH at *T* = 50 °C. By varying the etching time, the diameter of the nanochannels was adjusted. Etching times of 3.2, 5.6, 6.5, and 7.3 min resulted in nanochannels with average diameters of ∼80, ∼130, ∼150, and ∼170 nm, respectively. The fabricated etched ion-track membranes served as templates for the electrodeposition of Au nanowire networks.

(ii) For the electrodeposition of the nanowires, one side of the membrane was coated with a gold layer serving as a working electrode during the subsequent nanowire growth. First, a thin Au layer was sputtered using an Edwards Sputter Coater S150B (pressure 10^−1^ torr, current 30 mA, 200 s). This sputtered layer was then reinforced with an electrodeposited Au layer to provide a thicker and more stable support layer for the nanowire network. This Au back-electrode layer was fabricated by applying a galvanostatic electrodeposition process in a three-electrode configuration, at room temperature, applying 2 mA cm^−2^ current density using a commercial gold sulphite solution (AuSF, 15 g per L Au, METAKEM) electrolyte. This Au back-electrode acted as a working electrode during the subsequent nanowire electrodeposition step.

The electrodeposition of the nanowires was conducted at 60 °C, using a cyanide-based alkaline electrolyte, which contained 50 mM KAu(CN)_2_, 250 mM Na_2_CO_3_, and 1 vol% Dowfax 2A1 surfactant. Before the deposition process, the set-up was pre-heated for 1 h. The deposition was performed potentiostatically using a potentiostat Gamry 600+ and a three-electrode set-up, applying the voltages *U* = −1.1, −1.0, −0.9, −0.8, and −0.7 V *vs.* Ag/AgCl reference electrode (Sensortechnik Meinsberg GmbH, sat. KCl). A Pt spiral wire (Good-fellow, 99.99%) acted as the counter electrode.

(iii) To release the network from the PC membrane, the polymer was dissolved by rinsing the sample multiple times in dichloromethane.

The morphology and composition of the nanowires and networks were characterized by high-resolution scanning electron microscopy (HRSEM) and energy dispersive X-ray spectroscopy (EDX). SEM and EDX measurements were performed with a Zeiss Gemini 500 field emission microscope equipped with a Bruker EDX spectrometer. The nanowire crystallinity was analyzed by transmission electron microscopy (TEM) with a JEOL ARM200F TEM. For this, the nanowire network sample was immersed in isopropanol in an ultrasonic bath. Network fragments and individual nanowires were then drop-cast onto standard lacey carbon-supported copper TEM grids (Plano GmbH).

The ECSA measurements were conducted in 0.1 M H_2_SO_4_ solution, by running cyclic voltammetry (CV) with a scan rate of 100 mV s^−1^ in a commercial gas-tight cell (Redoxme AB). The Au NWNW sample acted as the working electrode. The counter electrode was a platinum auxiliary wire, and the reference electrode was an Ag/AgCl (3 M KCl) aqueous electrode. Before running the CV measurements, the electrolyte was purged with nitrogen for 30 min. The electrochemical catalytic activities of the nanowire networks and their stability and recyclability were investigated by measurements carried out using an alkaline 0.1 M KOH aqueous solution. For each sample, 20 CV cycles from −0.6 V to 0.8 V (*vs.* Ag/AgCl) were recorded (scan rate = 50 mV s^−1^). Then, a different concentration of methanol was added inside the 15 ml reactor, and CV cycles were recorded with the same scan rate and within the same potential range. All the CV measurements were carried out at room temperature (22 °C).

## Results and discussion

### Synthesis and characterization

**Fig. 2:**
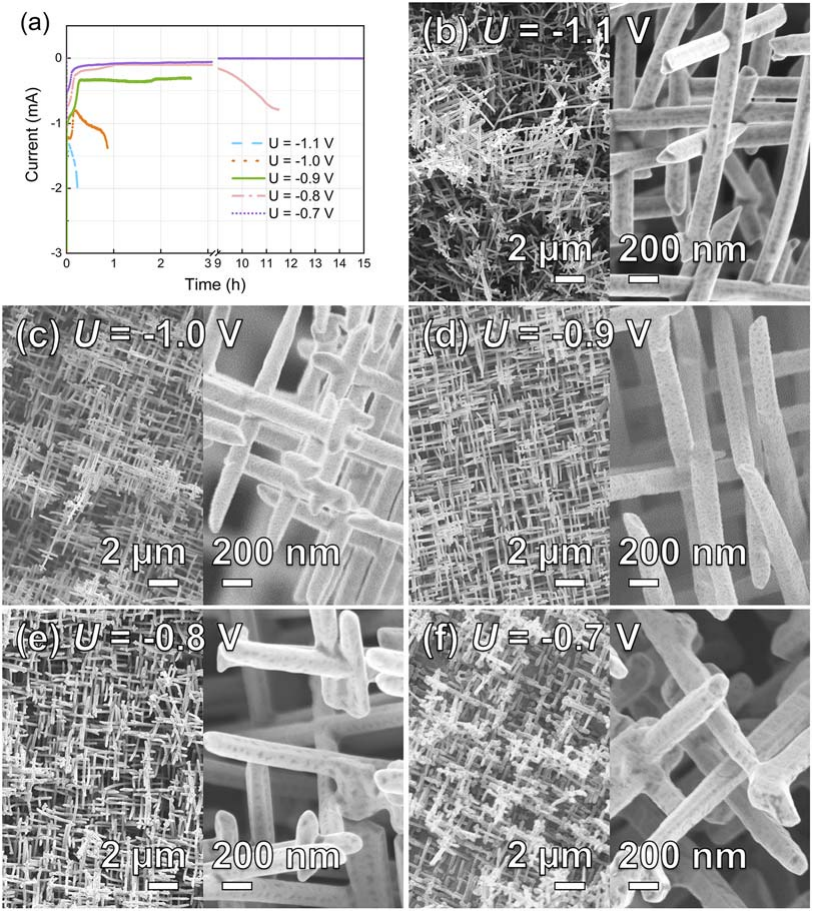
(a) Current *vs.* time (*I*–*t*) curves for various applied potentials recorded during the potentiostatic deposition of Au NWNWs, and the corresponding SEM images, (b) *U* = −1.1 V, (c) *U* = −1.0 V, (d) *U* = −0.9 V, (e) *U* = −0.8 V, (f) *U* = −0.7 V *vs.* Ag/AgCl. The large magnification SEM images reveal cylindrical monodispersed nanowires.

To find the optimal potential window for the homogeneous growth of Au NWNWs, the electrodeposition process was monitored *via* current *vs.* time (*I*–*t*) curves (Fig. 2(a)). Different potentials ranging from *U* = −1.1 V to *U* = −0.7 V (*vs.* Ag/AgCl), at 60 °C were applied. To allow direct comparison, all the etched ion-track templates had the same parameters, *i.e.* pore diameter of 150 nm and total nanochannel density of 4 × 10^8^ cm^−2^. The experimental results revealed a very strong influence of the deposition potential on both the Au NWNW growth rate and the resulting morphology. At high potentials, *e.g. U* = −1.1 V (blue dashed line), the growth was very fast. The current increased rapidly during the growth in the channels and even more rapidly after 13 min, indicating that some nanochannels were already completely filled and so-called caps formed on the membrane surface. This fast growth resulted in an inhomogeneous filling and thus poor interconnectivity, with a rather inhomogeneous and unstable network structure (Fig. 2(b)). At *U* = −1.0 V, the growth rate slightly decreased. Au nanowires were deposited in the channels for ∼1 h, and the overall homogeneity was improved (Fig. 2(c)). However, not all the channels were filled at the same rate, and thus some areas with fewer nanowires were observed. At lower potentials, namely *U* = −0.9 V and *U* = −0.8 V, the network homogeneity was significantly improved, resulting in a dense network structure. Cross-section SEM images of the Au NWNWs further demonstrated the homogeneity increase with decreasing the applied potential (see ESI Fig. S1†). However, the duration for complete channel filling became longer and longer. At *U* = −0.7 V, the template was not completely filled even after 15 h. At such long deposition times, a constant ion concentration in the electrolyte could not be guaranteed because the whole set-up was heated and the electrolyte solution evaporated over time. Based on these observations, an optimal deposition potential of *U* = −0.9 V was selected for the following experiments, which delivered an optimized morphology and good time efficiency.

From the SEM images, the nanowire diameter could be determined. By measuring the diameter of 100 wires, we calculated an average diameter of 148 ± 13 nm, *i.e.* a diameter spread distribution smaller than 10%. This was done for each sample, since the diameter of the wire determines the geometrical and electrochemically active surface area of the entire network. Their surface structure, in addition, can influence the catalytic activity. EDX analysis showed that the nanowires consisted of pure gold, and no impurities were detected, except a small peak for carbon (Fig. S2 in ESI†), where only Au and C peaks were visible, and the carbon was ascribed to the polymer residual left from the PC template removal.

**Fig. 3:**
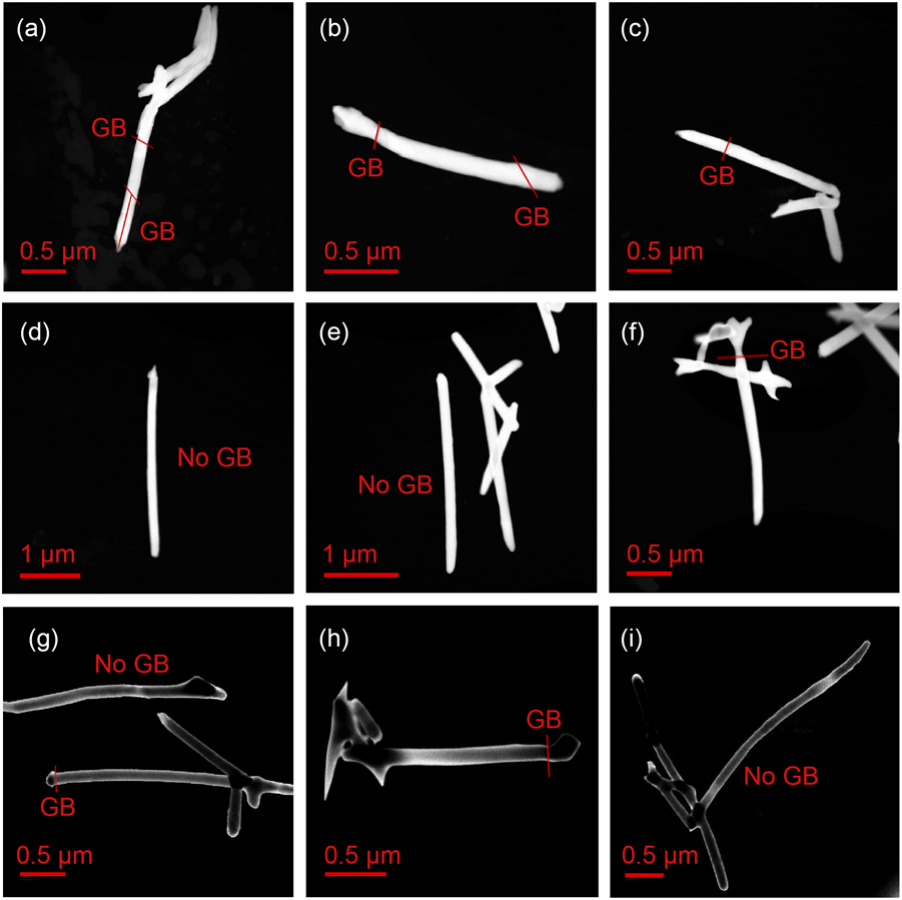
TEM dark-field images of fragments of Au nanowire networks deposited at (a–c) *U* = −1.0 V, (d–f) *U* = −0.9 V, which are high-angle annular dark-field images, and (g–i) *U* = −0.8 V *vs.* Ag/AgCl, are medium-angle annular dark-field images. The red lines mark the positions where grain boundaries (GB) were observed.

The crystallinity of the Au nanowires was analyzed by TEM. Fig. 3 shows representative TEM images of segments of the Au NWNW deposited at *U* = −1.0, −0.9, and −0.8 V *vs.* Ag/AgCl. By TEM dark-field imaging, grain boundaries (GB) were identified and marked with red lines in Fig. 3. The nanowires deposited at −1.0 V (Fig. 3(a)–(c)) were single crystalline with a length of about 0.5 to 1 μm, which indicated a bamboo-like crystalline structure. At less negative deposition potentials (*U* = −0.9 V in Fig. 3(d)–(f) and *U* = −0.8 V in Fig. 3(g)–(i)), the size of the grains was larger and the wires exhibited fewer grain boundaries, consistent with a smaller nanowire growth rate, as discussed above (Fig. 2(a)). It is also worth noting that no grain boundaries were found at the nanowire crossing junctions, so the presence of crossing nanochannels did not seem to affect the nanowire crystallinity.

### Electrochemical characterization

To study the electrochemical properties of these Au NWNWs for catalytic applications, a series of etched ion-track membranes were fabricated with the same nanochannel density and wire length, but with different average nanochannel diameters, although in the following nominal values are used to describe the characteristics of the different samples. In all cases, the applied potential during electrodeposition was *U* = −0.9 V *vs.* Ag/AgCl to guarantee homogeneous growth. Each sample was inserted into a three-electrode electrochemical cell to determine its ECSA, and was subsequently tested as an electrode during the well-known methanol oxidation reaction, carried out in an alkaline environment at room temperature. The performance of the networks was compared to a planar Au film that acted as a reference sample (details on this reference film are provided in ESI, Fig. S3†).

#### ECSA determination

**Fig. 4:**
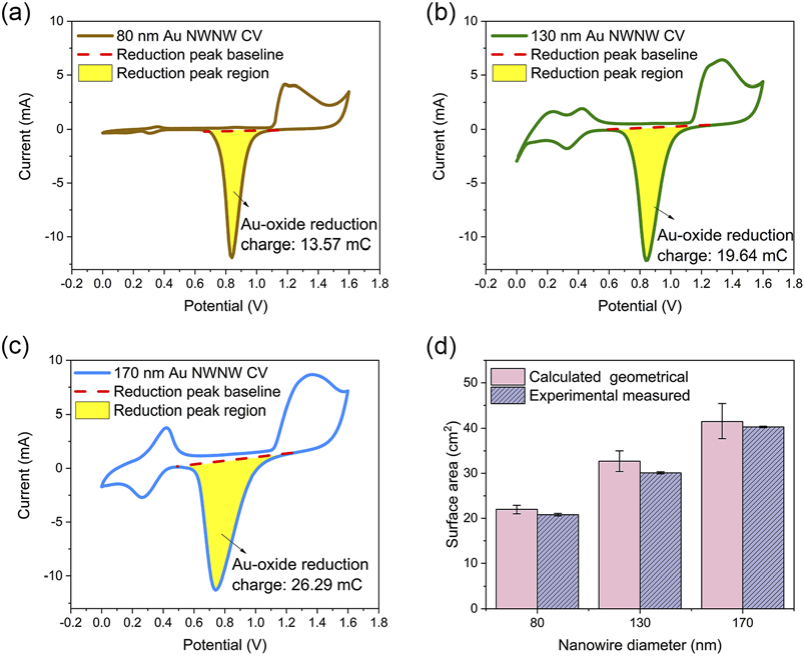
CVs recorded in 0.1 M H_2_SO_4_ for Au NWNWs of different nanowire diameters: (a) 80 nm, (b) 130 nm, and (c) 170 nm (room temperature, scan rate = 100 mV s^−1^). The red dashed line defines the baseline for the reduction peak integration, the yellow area represents the Au oxide reduction peak region providing the electrochemically active surface area. (d) Experimental (ECSA) and calculated (*S*_GEO_) surface area values of the Au NWNWs as a function of nanowire diameter.

The ECSA values were determined by integrating the faradaic charge transfer of the Au surface oxide reduction reaction.^50^ The voltammograms in Fig. 4 show the CVs recorded in 0.1 M H_2_SO_4_ solution. The anodic peaks located at 1.2–1.4 V (Fig. 4(a)–(c)) were attributed to the formation of Au oxide,^51,52^ while the current rising at 1.6 V represented the beginning of O_2_ evolution.^47^ The cathodic peak at 0.85 V represented the Au oxide reduction, with the peak area representing the total charge transfer during the reduction reaction. During the anodic scan, a monolayer of Au oxide formed on the Au NWNW surface, which was reduced during the cathodic scan. By integration of the reduction peak area and subtraction of the background, the total charge transfer could be determined.^47^ A reference value of the faradaic charge of a Au oxide monolayer per unit area was obtained by reference CV measurements under the same experimental conditions using the Au film on a 1 cm^2^ reference sample. By integrating the respective reduction peak, a reference charge-transfer value of *Q*_ref_ = 653 μC cm^−2^ was obtained (ESI, Fig. S4†).

Fig. 4(d) shows the ECSA values (purple bars) and *S*_GEO_ (pink bars) of three Au NWNWs (the error bars represent fluctuations from different CV cycles). The geometrical surface area (*S*_GEO_) of the NWNW samples was calculated by considering the nominal ion fluence of the PC foil, which was (2 ± 0.4) ×10^8^ ions per cm^2^, the length of the nanowires (determined from the height of the network and the 45° inclination angle of the wires), and the nanowire diameters measured by SEM, which were 86 ± 14, 131 ± 15, and 170 ± 17 nm (more information in ESI, Fig. S5†). The error bars of the calculated surface area included the uncertainties of the above parameters and accounted for the crossing points of nanowires caused by the stochastic distribution of the ions when irradiating the polymer template. Taking into account these uncertainties, for all nanowire diameters, ECSA and *S*_GEO_ were in very good agreement, indicating that the entire highly interconnected 3D Au NWNW contributed to the reaction.

#### Methanol oxidation reaction

**Fig. 5:**
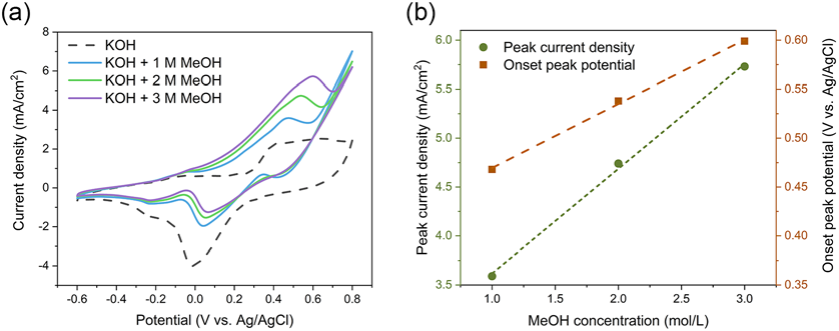
(a) CVs recorded in 0.1 M KOH with 0/1/2/3 M MeOH with scan rate 50 mV s^−1^, (b) anodic peak current density and onset potential as a function of the methanol concentration. The errors are accounted for the size of the symbols. Dash lines are the linear fitting of data points.

Next, the electrochemical activity of the Au NWNWS during the methanol oxidation reaction was studied. Fig. 5(a) shows CV cycles for 160 nm NWNW in 0.1 M KOH solution (black dashed lines) and in 0.1 M KOH + MeOH solution (solid lines). The respective current density values were determined by dividing the recorded current values by the nominal area of the sample (same as the area of the reference sample 1 cm^2^).

The methanol oxidation process involves two reactions: In the lower potential range, methanol is oxidized to formate, while in the higher potential region, formate is further oxidized to carbonate.^24^ In Fig. 5 (black dashed line), at *U* = 0.35–0.6 V, two anodic peaks could be superimposed into a plateau, corresponding to the formation of Au–OH and AuO, while the cathodic peak, located at *U* = 0.02 V, could be attributed to the stripping of Au–OH, and the reduction of AuO. The CV curve recorded in KOH electrolyte containing 1 M MeOH (blue solid line) exhibited one anodic peak at *U* = 0.46 V, which was the oxidation peak for methanol to formate on the Au surface,^53^ for which the peak current density was rather low, around 3.5 mA cm^−2^. When the methanol concentration increased to 2 M and 3 M, the oxidation peak current density increased accordingly to 4.7 and 5.7 mA cm^−2^. Fig. 5(b) also shows that the methanol oxidation peak current density increased proportionally to the methanol concentration.

Fig. 5(a) shows a shift of the anodic peak onset potential, which increased from 0.47 V to 0.6 V with the methanol concentration increasing. This may be caused by the increase in unoxidized organic residue absorbed on the surface, which required a higher potential for oxidation,^54^ or by an increase in the IR-drop.^55^ It is worth noticing that in spite of the high methanol concentrations, no saturation of the peak current density was reached.^54,55^

**Fig. 6:**
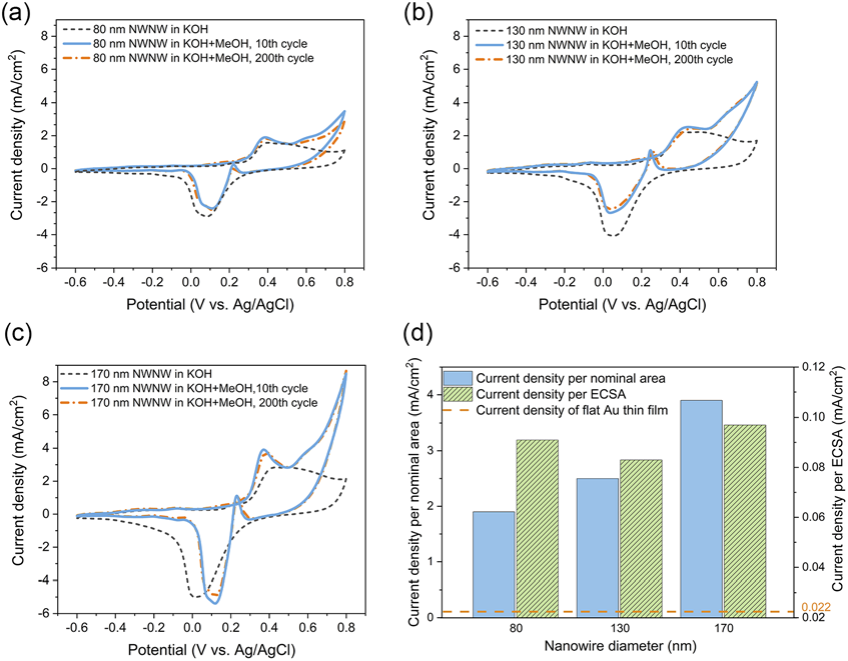
CVs recorded in 0.1 M KOH (black dash line) and in 0.1 M KOH + 0.5 M MeOH electrolyte including the 10th cycle (blue solid line), and the 200th cycle (orange dash-dot line) for (a) 80 nm, (b)130 nm, and (c) 170 nm. (d) Methanol electro-oxidation peak current density divided by nominal area (blue) and ECSA (shadowed green).

The influence of the nanowire diameter was investigated with a series of 3D NWNWs with nanowire diameters of 80, 130, and 170 nm, in 0.1 M KOH with and without 0.5 M MeOH. For the Au NWNW with 80 nm diameter nanowires (Fig. 6(a), solid blue line), the anodic peak of the methanol oxidation was located at *U* = 0.4 V with a peak current density of ∼1.9 mA cm^−2^, with the second anodic peak at *U* = 0.6 V for methanol oxidation on AuO, and formate oxidation to carbonate.^56^ With the nanowire diameter increasing, the anodic peak current density increased accordingly in the same nominal area. For each Au NWNW sample, 200 CV cycles in the KOH electrolyte with MeOH were run for 3 h. The CV curves for the 200th cycle were nearly the same as for the 10th cycle. Two major anodic and cathodic peaks were slightly shifted, whereby the change in the peak current density was smaller than 5%, and the peak potential was shifted only 0.02 V (Fig. 6(a)–(c)). These minor changes give evidence that during the overall 200 cycles, the reactions did not severely modify the samples and that the Au NWNW were stable units and thus have potential for long-term electrochemical performance.

Fig. 6(d) summarizes the current density values recorded for all the methanol oxidation peaks of the Au NWNW samples in reference to both the nominal area and ECSA. Compared with the planar Au reference sample in ESI Fig. S7,† the oxidation peak current density values per nominal area of the Au NWNW with nanowire diameters of 80, 130, and 170 nm were 86, 132, and 205 times higher, respectively. When divided by the measured ECSA of the Au NWNWs, the current density was still 4 times higher than for the flat film (see ESI Fig. S7†). The Au NWNWs were electrochemically more active in the catalytic reactions towards methanol electro-oxidation compared to the planar smooth sample. Thus, the large surface area of the 3D networks resulted in larger current densities, while the additional increase was attributed to the high electrochemical activity of the nanowire surface. The obtained values were comparable to those obtained by Graf *et al.* for nanoporous gold samples, which exhibited a peak current density of 0.042–0.127 mA cm^−2^ normalized to ECSA (in 1 M KOH + 1 M CH_3_OH electrolyte).^7^

**Fig. 7:**
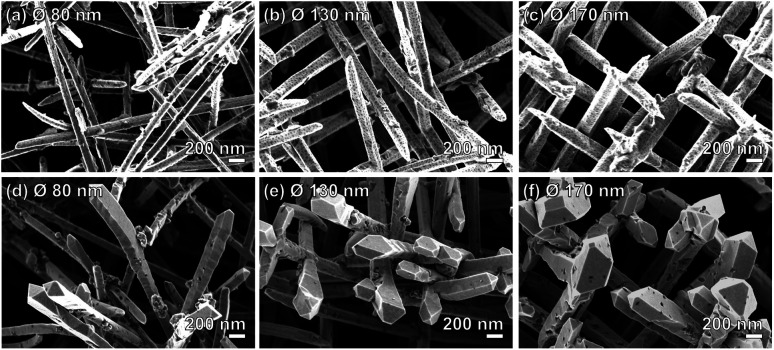
SEM images of Au NWNW deposited at *U* = −0.9 V *vs.* Ag/AgCl with different nanowire diameters, (a) and (d) 80 nm, (b) and (e) 130 nm, (c) and (f) 170 nm, before (a–c) and after (d–f) 200 CV cycles during methanol oxidation reaction measurements.

The geometry of the samples allowed us to investigate possible morphological changes of the Au NWNWs that may have occurred during the CV measurements, by characterizing the samples by SEM before and after the methanol electro-oxidation. The SEM images in Fig. 7 reveal that the NWNWs maintained their original 3D interconnected structure throughout the 3 h long process, while the individual nanowires exhibited clear changes in morphology, especially at the upper tips. At the nanowire tips, facetted crystals were formed with larger diameters compared to the initial wire diameters. Such morphology changes have been reported before, *e.g.* by Arán-Ais *et al.*, who showed that both the shape and morphology of Pt nanoparticles were drastically degraded after 1000 CV cycles in a 0.5 M H_2_SO_4_ solution.^57^ Surface restructuring was also reported by Cui *et al.* for porous PtCu nanotubes after 250 cycles in 0.1 M HClO_4_ solution.^58^

In our case, since the long nanowire sections (length ∼ 27–28 μm) below the tips remained unchanged, the overall surface area change was smaller than 4.5%. Thus, it should be emphasized that the catalytic performance of the Au NWNWs was not affected by the observed changes in the tip morphology, as evidenced by the excellent stability of the CV curves between the 10th and the 200th cycles.

## Conclusions

Au NWNWs with various nanowire diameters were successfully synthesized by electrodeposition in etched ion-track PC templates. The deposition potential had a direct influence on the nanowire growth rate, nanowire morphology, and overall homogeneity of the network. For the potentiostatic deposition of the Au NWNWs, a voltage of *U* = −0.9 V *vs.* Ag/AgCl yielded an excellent compromise between the optimal network properties and growth duration. The ECSA of the NWNWs was determined by CV measurements of the Au oxide reduction, which were in excellent agreement with the calculated surface areas *S*_GEO_ based on geometrical considerations. The electrochemical performance of the Au NWNWs was investigated by CV during methanol oxidation reactions, showing that the catalytic activity was higher than that of a flat reference sample and was stable during long-term testing. The methanol oxidation peak current density increased with increasing the methanol concentration in the electrolyte, which also increased with the larger ECSA, demonstrating that our Au NWNW has large potential as a methanol fuel cell catalyst. Its major advantage comes from the large surface area of the NWNW structure, which highly promotes the reaction rate. Another important phenomenon is that the optimized Au nanowire networks exhibited high geometrical stability, maintaining their original structure during long-term CV measurements over several hours. Thanks to their large surface areas and geometry enabling sufficient access of the electrolyte and reactants to the surface, the Au NWNW provided current densities up to 200 times higher than the flat Au reference. During 200 cyclic voltammetry reaction cycles, the current density kept rather stable, dropping by only 5% at the beginning and remaining stable for 3 h after that. The overall excellent performance demonstrates the enormous potential of nanostructured Au electrodes for methanol fuel cells, as well as for further electrocatalytic reactions, such as ethanol oxidation, CO_2_ reduction, and nitrogen reduction to ammonia.^59–61^

## Author contributions

M. Li and M. E. T.-M. designed the experiments. M. Li carried out the experiments, analyzed the data, and prepared the manuscript. I. S., N. U., and M. F. P. W. helped with sample preparation and SEM studies. W. S. performed the TEM analysis. The manuscript has been discussed by all authors. M. Li, C. T., and M. E. T.-M. discussed the data and finalized the manuscript.

## Conflicts of interest

The authors declare no conflicts of interest.

## Supplementary Material

RA-013-D2RA08035D-s001
