# Supplementary material for: Three-dimensional free-standing gold nanowire networks as a platform for catalytic applications
Source: RSC Adv. 2023 Feb 3;13(7):4721–8. doi: 10.1039/d2ra08035d (PMC9897260; doi:10.1039/d2ra08035d)
Supplement: RA-013-D2RA08035D-s001 [file RA-013-D2RA08035D-s001.pdf]

Electronic Supplementary Material (ESI) for RSC Advances.
This journal is © The Royal Society of Chemistry 2023

Supporting Information

Cross-section imaging of Au NWNWs

Figure S1 shows cross-section SEM images of Au nanowire networks deposited at different potentials: At $U = -1.1$ V the network structure is denser at the bottom, but gets looser towards the upper part, which is a possible hint that not all nanochannels were filled during electrodeposition (Figure S1 (a)). The total height of the network differs at different areas, the largest difference reaching ~15 µm (higher parts are indicated by arrows). Given the fact that the whole template is only 30 µm thick, this height difference is 50%. When the deposition potential is decreased to $U = -1.0$ V, the Au nanowires grow almost to the full length of the template (Figure S1 (b)), with the height difference at different spots ranging from 5 µm to 10 µm. The network structure is very well interconnected. For the Au nanowire network deposited at $U = -0.9$ V, the height fluctuations are below 5 µm, and the network structure is very homogeneous and dense (Figure S1 (c)). The sample is mechanically stable, can easily cut without fragmentation and shows high ductility

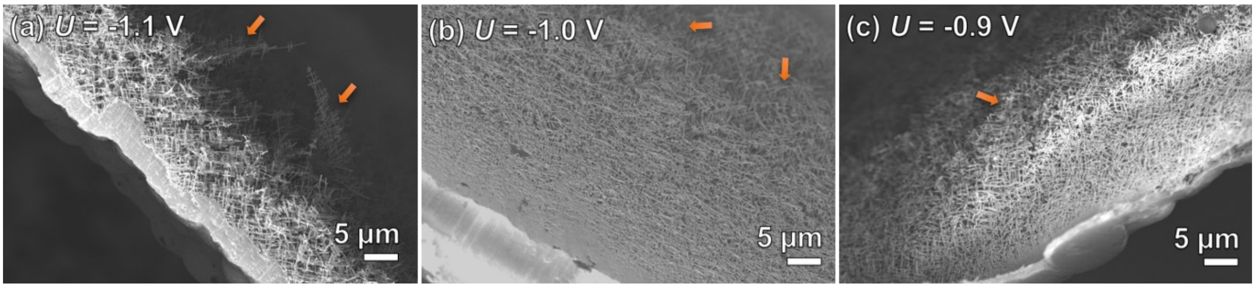

Figure S1. SEM images of cross-sections of Au nanowire network deposited at (a) $U = -1.1$ V, (b) $U = -1.0$ V (c) $U = -0.9$ V vs. Ag/AgCl.

EDX Spectrum of Au NWNW

The EDX spectrum of a Au NWNW deposited at U = -0.9 V vs. Ag/AgCl, shows the pronounced signal from Au originating from the nanowires and the back-electrode layer (Figure S2). . The small peak ascribed to carbon possibly comes from the residual polymer from the template remaining on the nanowire surface.

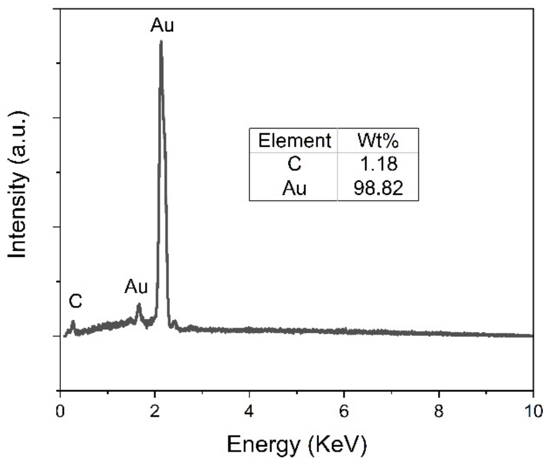

Figure S2. EDX spectrum of Au NWNW deposited at U = -0.9 V vs. Ag/AgCl.

Planar Au reference sample

The planar Au reference sample (5 µm thick) was galvanostatic electrodeposited in a three-electrode setup, using a PC polymer foil as substrate. The deposition process was conducted at room temperature applying 2 mA/cm^2 current density for 1 h, using a commercial gold sulfite (AuSF, 15 g/L Au, METAKEM) electrolyte. As shown by SEM (Figure S4), the sample surface is very smooth, only at very high magnification some small structures are visible.

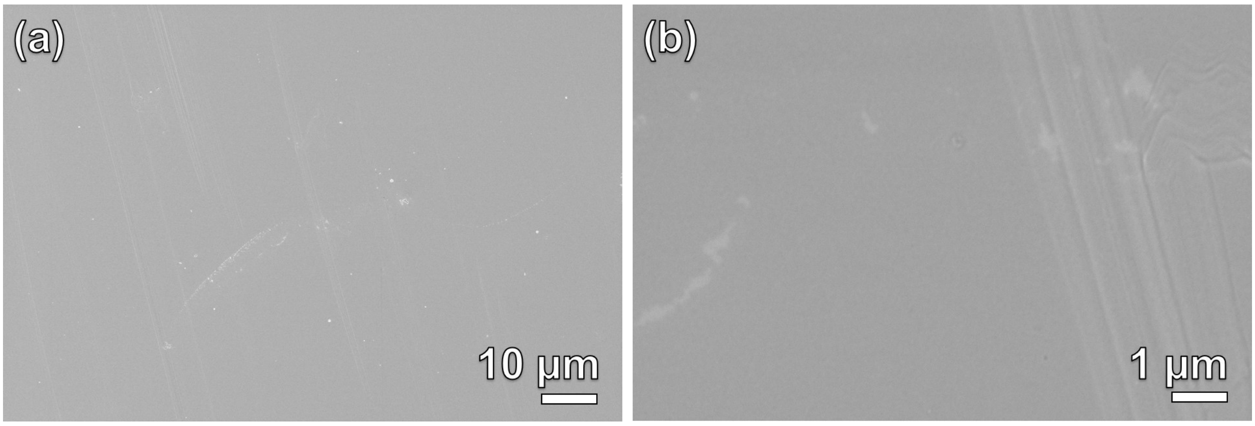

Figure S3. SEM images of the planar Au reference sample

The measurement of the electrochemically active surface area of the flat Au reference sample was conducted at room temperature, with the same experimental setup as used for the Au NWNWs. By integrating the area of the reduction peak (Figure S5) a total charge of 653 µC was deduced for a monolayer of AuO reduction. The exposed area of the flat Au sample was 1 cm^2, thus the reference value for the charge transfer for a monolayer of AuO reduction is 653 µC/cm^2.

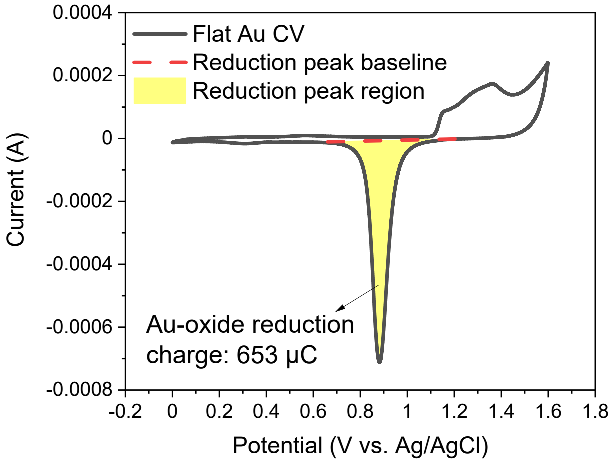

Figure S4. CV recorded in 0.1 M H_2SO_4 for a 1cm^2 flat Au reference sample.

Distribution of gold nanowire diameters

From each Au NWNW sample, the diameters of 100 wires were measured. From the corresponding size distributions presented in figure S3, the mean values were deduced.

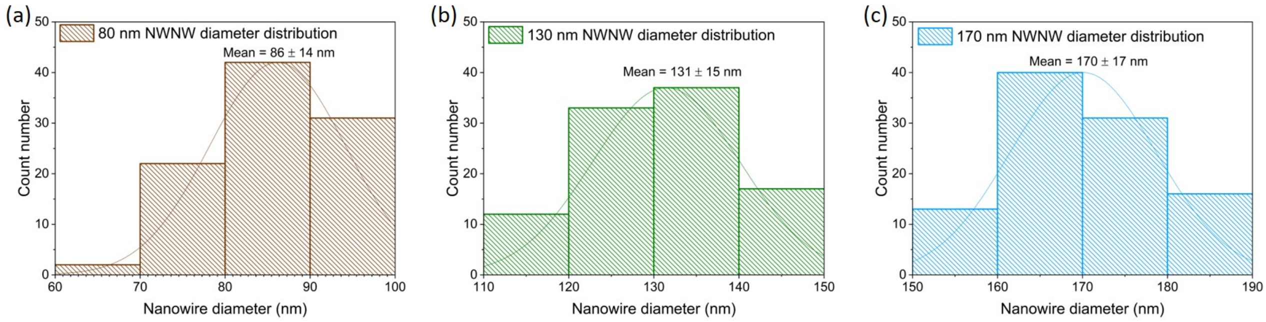

Figure S5. Nanowire diameter distribution chart of Au NWNWs

Planar Au reference sample towards methanol electro-oxidation

Figure S6 shows CV cycles for the flat reference samples and for NWNW with different wire diameters recorded in a 0.1 M KOH solution (black dashed lines) and in a 0.1 M KOH + 0.5 M MeOH solution (solid lines), respectively. The respective current density values were calculated determined by dividing the recorded current values by the nominal area.

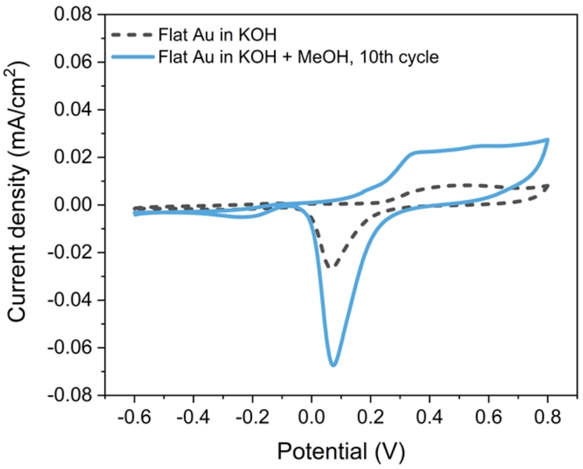

Figure S6. CVs recorded in 0.1 M KOH (black dash line) and in 0.1 M KOH + 1 M MeOH (blue solid line) electrolyte including the 10th cycle (blue solid line), for planar Au reference sample.

Au NWNW catalytic reactions towards methanol electro-oxidation

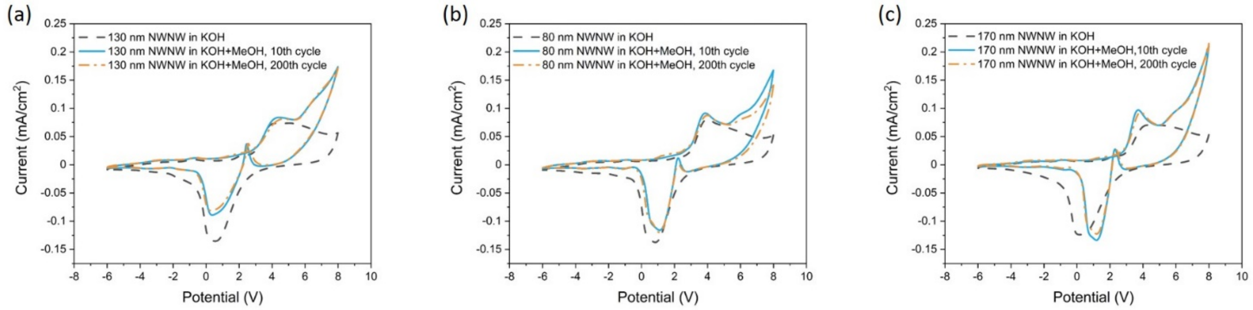

Figure S7. CVs recorded in 0.1 M KOH (black dash line) and in 0.1 M KOH + 1 M MeOH electrolyte including the 10th cycle (blue solid line), and the 200th cycle (orange dash-dot line) for Au NWNW with average nanowire diameter (a) 80 nm, (b)130 nm, and (c) 170 nm.
